# TLR4 gene polymorphisms in Egyptian vitiligo patients: insights into emerging association with clinical activity, family history, and response to therapy

**DOI:** 10.1186/s43141-021-00218-y

**Published:** 2021-09-01

**Authors:** Maha Abdelsalam, Sherihan H. Allam, Marwa Zohdy, Hend Magdy, Maged Mostafa

**Affiliations:** 1 Immunology Unit, Clinical Pathology Department, Faculty of Medicine, Mansoura University, Mansoura, Egypt; 2 Department of Immunology, Egypt Center for Research and Regenerative Medicine (ECRRM), Cairo, 11517 Egypt; 3 Dermatology Department, Faculty of medicine, Mansoura University, Mansoura, Egypt; 4 Public Health & Community Department, Faculty of medicine, Mansoura University, Mansoura, Egypt

**Keywords:** TLR4, Vitiligo, Autoimmunity, Innate immunity, Egyptian

## Abstract

**Background:**

Vitiligo is a common pigmentary disorder in which autoimmunity has been suggested to play an important role. Toll-like receptor (TLR) family are recognized different molecular structures expressed on immune cells and have been implicated in a number of autoimmune diseases (AIDs) such as vitiligo. The purpose of this study was to investigate the possible association between TLR4 gene polymorphisms: *rs11536858*, *rs1927911*, *rs1927914* in Egyptian vitiligo patients and their clinical data, their response to therapy. Using PCR-RFLP for TLR4 gene polymorphisms (*rs11536858*, *rs1927911*, and *rs1927914*), both alleles and genotypes were determined after extraction of DNA in a case-control study of 100 vitiligo Egyptian patients and 100 matched age and sex controls.

**Results:**

The distribution of the protective *CT* genotype of *rs1927914* was higher in the control group. After dividing both patients and controls into 2 age groups (below 18 and above 18 years), no significant associations between the genotypes of the selected TLR4 SNPs and the demographic and clinical data of the vitiligo patients in group 1 (below 18 years) were observed. For group 2 (above 18 years), also no significant associations were found except for the association between the *CC* genotype of *rs1927914* and psychiatric trauma, from one side, and between the *CT* genotype of *rs1927911* and alopecia, from the other side. The association between combined genotypes and the risk of vitiligo showed either higher frequency in patients (risky), or controls (protective), and some equal frequencies (non-significant). The association between haplotypes and risk of vitiligo in patients’ group revealed the highest frequency for the risky *ATT* and the least frequency for *ATC* haplotypes. In control group, the protective *GCT* haplotype showed the highest frequency while the *GTC* and *GCC* showed the least frequency. No significant correlations of haplotypes with clinical and demographic data of selected patients’ group were observed apart from that between *ACC* haplotype and family history of AIDs and between *ATT* haplotype and remission after phototherapy.

**Conclusions:**

The significant relationship between TLR4 gene polymorphisms and vitiligo patients charcteristics clarify the role of innate immunity in pathogensis of vitiligo and its effect on the used therapies.

## Background

Vitiligo is a common skin disorder, affecting the epidermis and the hair follicles, characterized by the presence of depigmented patches due to destruction of melanocytes. Its exact etiology is not well known [1]. There are many potential pathophysiological theories involving autoimmune, neural, autocytotoxic, biochemical, oxidative stress, melanocytorrhagy, and decreased melanocyte survival. Autoimmune theory is more prominent in generalized vitiligo, which is considered a complex disorder involving combined pathogenic effects of multiple susceptibility genes and unknown environmental factors that lead to the autoimmune destruction of melanocytes [2].

The innate immunity role in vitiligo is evident by the infiltration of natural killer (NK) cells which show 5-fold increase in lesional skin of vitiligo patients [3]. Toll-like receptors (TLRs) are arms of innate immunity, first reported in humans in 1998, that can recognize pathogen-associated molecular patterns (PAMPs) present in microbes [4]. TLRs act either by binding to lipoprotein and peptidoglycan (TLR2, 4, 5, 6, and 11) or by identifying single-strand RNA and double-strand RNA which are related to viral and bacterial nucleic acids (TLR3, 7, 8, and 9). After their stimulation by ligand binding, they participate in the killing of a pathogen by inflammatory cytokines and co-stimulatory gene expression [5].

Single-nucleotide polymorphism (SNP) genotyping technologies study the effect of amino acid alteration, which affects the function of the protein. SNPs in promoter regions change the transcription factor binding motifs that lead to suppression of the wild-type transcript and change the efficiency of enhancer or repressor elements, or add an alternative translation initiation codon [6].

There is a lack of research studies on TLRs gene polymorphisms in Egyptian vitiligo patients. The aim of this study is to identify if there is an association between TLR4 polymorphisms; namely *rs1927914*, *rs11536858*, and *rs1927911* genotypes; and the risk of vitiligo in Egyptian patients, their clinical data, and their response to therapy.

## Methods

This work was done as a case-control study conducted upon 100 clinically diagnosed vitiligo patients aged between 5 and 67 years with mean of 33.23 (SD = 16.98); they included 44 males (44%) and 56 females (56%). The patients were selected from inpatient and outpatient clinic of the Dermatology Department. Age and sex matched 100 apparently healthy subjects were selected to act as a control group. Their mean age ranged from 5 to 66 years with mean of 33.86 (SD = 15.923); they were 50 males (50%) and 50 females (50%). A written informed consent was taken from patients and controls. The study was approved by the responsible ethical committee of the research institute.

All vitiligo cases were subjected to: detailed history taking and clinical assessment: history included personal data, course and duration of the disease, history of psychic trauma, history of other skin or medical diseases, and family history of vitiligo or other AIDs. Thorough clinical examination included the exact site of the lesion, type of vitiligo either segmental or nonsegmental, vitiligo disease activity (VIDA) score [7], and response to the specific therapy.

### Genotyping

**Fig. 1:**
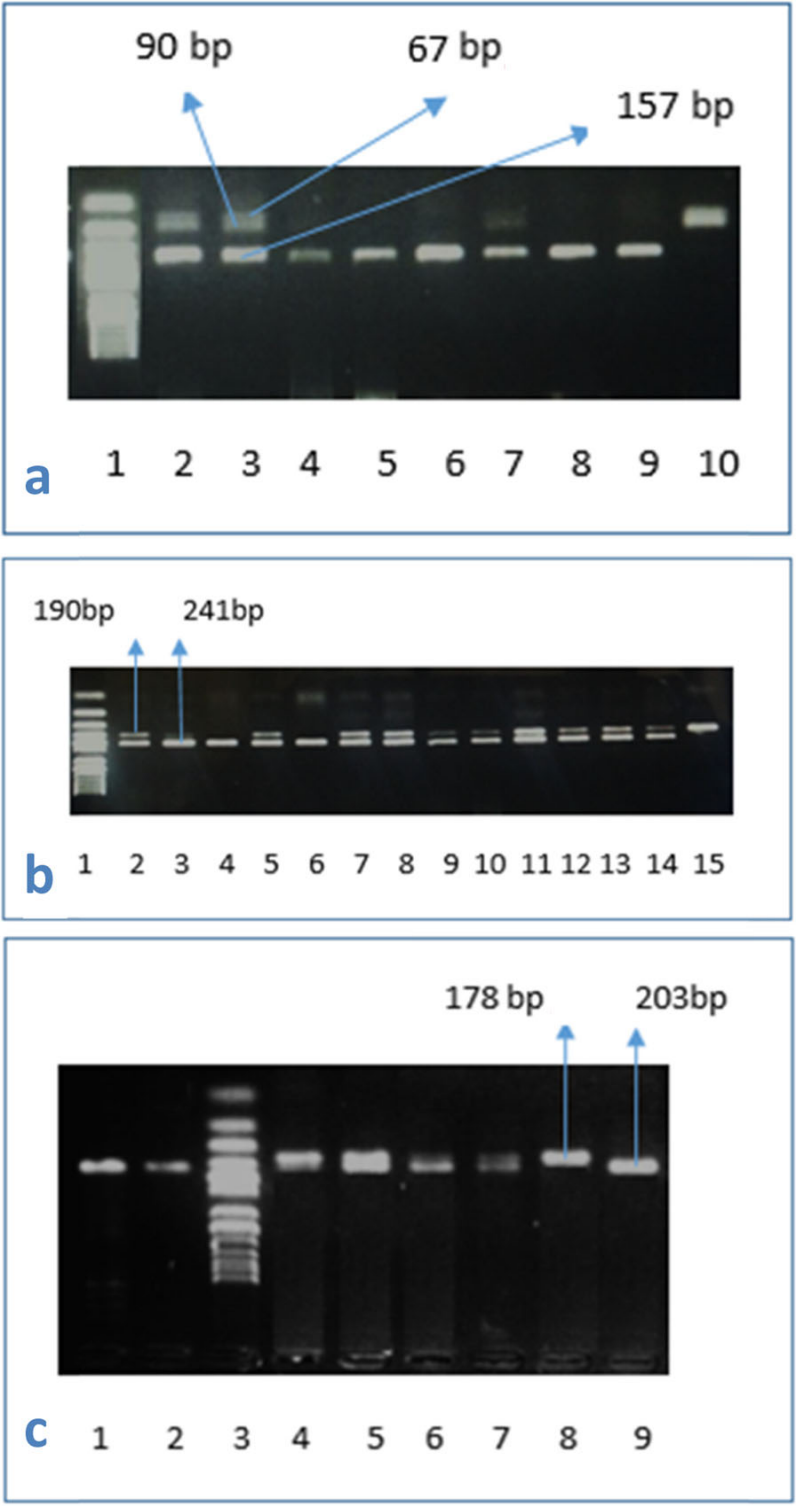
**A***rs1927914*, PCR product size was 157 bp. Restriction enzyme was SphI. Lane 1: ladder Lane 2,3: *CT (157,90,67)* Lanes 4–9: *TT (157)* Lane 10: *CC* (*90*,*67*). **B** *rs11536858*, PCR product size was 241 bp, restriction enzyme KpnI. Lane 1: Ladder Lanes 2, 5, 7–14: *AG* (*241*, *190.51*). Lanes 3, 4, 6: *AA* (*241*). Lane 15: *GG* (*190*,*51*). **C** *rs1927911*, PCR product size was 203 bp, restriction enzyme was StyI. Lanes 1, 2, 9: *TT* (*203 bp*). Lane 3: Ladder Lanes 4–7: *CT* (*203*, *178*, *25*). Lane 8: *CC* (*178*, *25*)

Whole blood samples were collected using EDTA tube and preserved at − 20 °C until the time of extraction. The DNA was extracted using whole blood purification kit (cat number: K0781; Thermo Scientific, Lithuania). The polymerase chain reaction-restriction fragment length polymorphism (PCR–RFLP) was the adopted technique for studying the selected SNPs as mentioned in previous studies for *rs11536858* (now merged into *rs10759931*) [8], *rs1927911* [5], and *rs1927914* [9]. The final PCR reaction volume was 25 μl: each primer 0.5 μl, H_2_O 4.0 μl, master mix (Thermo Fischer Scientific, USA) 15.0 μl, and extracted DNA 5 μl. To check the reaction products, we mixed 5 μl with 2 μl of loading buffer and migrated on 2% agarose gel. The volume for RFLP reaction was 30 μl: nuclease-free water 17 μl, PCR products 10 μl, restriction enzyme 1 μl, and 10× buffer 2 μl. All restriction enzymes used are thermally inactivated. The primers, restriction enzymes, and RFLP product sizes are explained in Table 1. After completion of digestion of amplicons with the selected restriction enzymes, the resultant fragments are resolved by gel electrophoresis as shown in Fig. 1 A, B, and C for rs1927914, rs11536858, and rs1927911 SNPs respectively.

**Table 1:** Details of RFLP products used in the study

SNP ID	Primers (5′–3′)	Restriction enzyme	Cat NO	RFLP product size
rs10759931	F: ATAACCTCAGTGGGCTCTGG R: ATGTTCTGGCATCTGGGAAG	KpnI	FD0524	AA = 241 AG = 241, 190, 51 GG = 190, 51
rs1927911	F: TCACTTTGCTCAAGGGTCAA R: AAACCTGCATGCTCTGCAC	StyI	ER0411	TT = 203 TC = 203, 178, 25 CC = 178, 25
rs1927914	F: ACAAAATGGTCCCTCACAGC R: TGGAAAGTAGCAAGTGCAATG	SphI	ER0601	TT = 157 TC = 157, 90, 67 CC = 90, 67

### Statistical analysis

All data was presented and tabulated using Excel program (Microsoft office 2016). SPSS version 25 (Statistical Package for Social Science) (SPSS, IBM, Chicago, III, USA) was used for statistical analysis. Frequency and percentage were used to express the qualitative data while mean and standard deviation were used for quantitative data. To find the association between variables of qualitative data, Chi-square and Fisher exact tests were used. To find the relation between non-parametric data, the one-way ANOVA test was used. The Hardy-Weinberg equilibrium was applied on all control group and the deviations were determined using the Chi-squared test. Odds ratio and 95% confidence interval were calculated. *p* value < 0.05 was considered significant. Haplotypes and linkage disequilibrium (LD) were detected using Haploview program version 4.2. GenAlEx version 6.5 was used to calculate pairwise FST statistics [10, 11].

## Results

Concerning the demographic and clinical data of the studied groups, both patients and controls were unrelated and selected randomly from Dakahleya Governorate, Egypt. The Hardy Weinberg equation was applied to control group. The TLR4 studied genotypes were independent (*p* > 0.05). The patients and controls were divided according to age to two groups. Group 1 less than 18 years old with 24 patients and 19 controls. Group 2 more than 18 years old with 76 patients and 81 controls. The genotype analysis and the association of different genotypes with patient data were done separately for both groups, while the haplotype and combined genotype analysis were done on all patients and controls (Table 2).

**Table 2:** Demographics characteristics of vitiligo patients

		Case	Control
Age/Years (MEAN ±SD) Group 1 (< 18 years) (MEAN ± SD) Group 2 (> 18 years) (MEAN ± SD)		33.23 ± 16.98 11.87 ± 4.14 39.97 ± 13.54	33.86 ± 15.9 12.37 ± 4.52 38.90 ± 13.18
Age of Onset/ Years (MEAN ± SD)		26.04 ± 14.83	
Gender	Male (*N*/%)	45/45%	
	Female (*N*/%)	55/55%	
Course of disease	Regressive (*N*/%)	6/6%	
	Progressive (*N*/%)	90 /90%	
	Stable (*N*/%)	4/4%	
Psychic trauma	Yes (*N*/%)	26 /26%	
	No (*N*/%)	74/74%	
Autoimmune diseases (AIDs)	Yes (*N*/%)	0/0%	
	No (*N*/%)	100/100%	
Alopecia	Yes (*N*/%)	14/14%	
	No (*N*/%)	86/86%	
Family history	Yes (*N*/%)	8/8%	
	No (*N*/%)	92/92%	
Family history of AIDs	Yes (*N*/%)	9/9%	
	No (*N*/%)	91/91%	
Remission	Yes (*N*/%)	63/63%	
	No (*N*/%)	37/37%	
Phototherapy	Yes (*N*/%)	99/99%	
	No (*N*/%)	1/1%	
Type	Segmental (*N*/%)	17/17%	
	Non-Segmental (*N*/%)	83/83%	
VIDA score	0	22/22%	
	1	13/13%	
	2	14/14%	
	3	15/15%	
	4	36/36%	

*SD* standard deviation

Tables 3 and 4 show the distribution of the genotypes and alleles of the selected SNPs in both patients and controls for both age groups. The only significant relation even after Bonferroni correction was applied, the *CT* genotype of *rs1927914* and was higher in control group (OR = 0.32, CI = 0.13–0.76, *p* = 0.01 and pc = 0.03). No other significant relations have been found for other alleles and genotypes (*p* > 0.05).

**Table 3:** Distribution of TLR4 alleles and genotypes Vitiligo patients and healthy controls (group 1: below 18 years)

TLR4 polymorphisms	Case (*N*—24) *N* (%)	Control (*N* = 19) *N* (%)	OR (95% CI)	p/pc
*rs1927914* (*C*/*T*)				
*CC*	3 (12.5)	0 (00.0)	1.91 (1.41–2.56)	0.24/NS
*CT*	3 (12.5)	4 (21.1)	0.54 (0.10–2.75)	0.68/NS
*TT*	18 (75.0)	15 (78.9)	1.25 (0.29–5.27)	0.76/NS
*C*	9 (18.7)	4 (10.5)	1.96 (0.55–6.95)	0.37/NS
*T*	39 (81.3)	34 (89.5)	0.51 (0.14–1.81)	0.37/NS
*rs1927911* (*C*/*T*)				
*CC*	4 (16.7)	2 (10.5)	1.70 (0.28–10.4)	0.68/NS
*CT*	15 (62.5)	16 (84.5)	0.31 (0.07–1.38)	0.12/NS
*TT*	5 (20.8)	1 (5.30)	0.21 (0.02–1.99)	0.21/NS
*C*	23 (48.0)	20 (52.6)	0.82 (0.35–1.94)	0.66/NS
*T*	25 (52.0)	18 (47.4)	1.21 (0.52–2.83)	0.66/NS
*rs11536858* (*A*/*G*)				
*AA*	5 (20.8)	5 (26.3)	0.74 (0.18–3.05)	0.67/NS
*AG*	17 (70.8)	9 (47.4)	2.69 (0.77–9.51)	0.12/NS
*GG*	2 (8.30)	5 (26.3)	0.93 (0.39–2.21)	0.21/NS
*A*	27 (54.6)	19 (50.0)	1.29 (0.54–3.02)	0.56/NS
*G*	21 (45.4)	19 (50.0)	0.78 (0.33–1.83)	0.56/NS

*OR* odds ratio, *95*%*CI* 95% confidence interval

Significant p/pc values if **<** 0.05, *pc* Bonferroni corrected *p* value (number of comparison × *p* value). *NS* non-significant

Fisher’s exact: no significant relations have been found between the alleles and genotypes (*p* > 0.05)

**Table 4:** Distribution of TLR4 alleles and genotypes vitiligo patients and healthy controls (group 2: above 18 years)

TLR4 polymorphisms	Case (*N*—76) *N* (%)	Control (*N* = 81) *N* (%)	OR (95% CI)	p/pc
*rs1927914* (*C*/*T*)				
*CC*	12 (15.8)	7 (8.6)	1.98 (0.74–5.34)	0.17/NS
*CT*	8 (10.5)	22 (27.2)	0.32 (0.13–0.76)	0.01/0.03
*TT*	56 (73.7)	52 (64.2)	0.64 (0.32–1.27)	0.20/NS
*C*	32 (21.0)	36 (22.2)	0.93 (0.55–1.60)	0.80/NS
*T*	120 (79.0)	126 (77.8)	1.07 (0.62–1.83)	0.80/NS
*rs1927911* (*C*/*T*)				
*CC*	13 (17.1)	26 (32.1)	0.44 (0.21–0.93)	0.03/NS
*CT*	50 (65.8)	42 (51.9)	1.79 (0.94–3.40)	0.08/NS
*TT*	13 (17.1)	13 (16.0)	0.93 (0.39–2.15)	0.44/NS
*C*	76 (50.0)	94 (58.0)	0.72 (0.46–1.13)	0.15/NS
*T*	76 (50.0)	68 (42.0)	1.38 (0.88–2.16)	0.15/NS
*rs11536858* (*A*/*G*)				
*AA*	19 (25.0)	20 (24.7)	1.02 (0.49–2.09)	0.96/NS
*AG*	45 (59.2)	49 (60.5)	0.94 (0.50–1.80)	0.87/NS
*GG*	12 (15.8)	12 (14.8)	0.93 (0.39–2.21)	0.87/NS
*A*	83 (54.6)	89 (54.9)	0.99 (0.63–1.54)	0.95/NS
*G*	69 (45.4)	73 (45.1)	1.01 (0.64–1.58)	0.95/NS

*OR* odds ratio, *95*%*CI* 95% confidence interval

Significant *p*/pc values if **<** 0.05, *pc* Bonferroni corrected *p* value (number of comparison × *p* value). *NS* non-significant

Fisher’s exact: the *CT* genotype of *rs1927914* was significantly higher in control group

Table 5 shows the relation between the genotypes of the selected TLR4 SNPs and the demographic and clinical data of the vitiligo patients in group 1 of vitiligo patients. No significant associations were detected. Table 6 shows the relation *n* between genotypes and the patient data in group 2 of vitiligo patients. No significant associations were found except for the association between the *CC* genotype of *rs1927914* and psychiatric trauma (*p* 0.02) and between the *CT* genotype of *rs1927911* and alopecia (*p* 0.03).

**Table 5:** Relation of TLR4 genotypes versus demographic, clinical, and outcome data in vitiligo group 1 (below 18 years) patients

SNPs		*rs1927914*			*rs1927911*			*rs11536858*		
		*CC* *N*/*%*	*CT* *N*/*%*	*TT* *N*/*%*	*CC* *N*/*%*	*CT* *N*/*%*	*TT* *N*/*%*	*AA* *N*/*%*	*AG* *N*/*%*	*GG* *N*/*%*
Sex	M/F	0.20/0.03	0.20/0.15	0.60/0.81	0.10/0.15	0.80/0.69	0.10/0.15	0.20/0.24	0.70/0.57	0.10/0.18
	*p*	0.13	0.66	0.15	1.0	0.53	1.0	1.0	0.48	1.0
Course	Regressive/Progressive	0.00/0.13	0.00/0.13	1.00/0.73	0.00/0.17	1.00/0.61	0.00/0.22	0.00/0.22	1.00/0.70	0.00/0.09
	*p*	1.0	1.0	1.0	1.0	1.0	1.0	1.0	1.0	1.0
Psychiatric trauma	+/−	0.33/0.09	0.00/0.14	0.67/0.76	0.00/0.19	0.67/0.62	0.33/0.19	0.00/0.24	1.00/0.67	0.00/0.09
	*p*	1.0	1.0	1.0	1.0	1.0	0.52	1.0	0.53	1.0
Alopecia	+/−	1.00/0.09	0.00/0.13	0.00/0.78	0.00/0.17	1.00/0.60	0.00/0.22	0.00/0.22	1.00/0.69	0.00/0.09
	*p*	0.13	1.0	0.25	1.0	1.0	1.0	1.0	1.0	1.0
FH of vitiligo	+/−	0.25/0.10	0.25/0.10	0.50/0.80	0.25/0.15	0.50/0.65	0.25/0.20	0.00/0.25	1.00/0.65	0.00/0.10
	*p*	0.44	0.44	0.25	0.54	0.62	1.0	0.54	0.28	1.0
FHAIDs	+/−	0.00/0.14	0.50/0.09	0.50/0.77	0.50/0.14	0.00/0.68	0.50/0.18	0.00/0.23	1.00/0.68	0.00/0.09
	*p*	1.0	0.24	0.45	0.31	0.13	0.38	1.0	1.0	1.0
Remission	+/−	0.29/0.06	0.14/0.12	0.57/0.85	0.43/0.06	0.43/0.71	0.14/0.24	0.29/0.18	0.57/0.77	0.14/0.06
	*p*	0.19	1.0	0.31	0.06	0.36	1.0	0.61	0.37	0.51
Type	Segmental/Nonsegmental	0.00/0.15	0.00/0.15	0.00/0.30	0.00/0.20	0.75/0.60	0.20/0.20	0.00/0.25	1.00/0.65	0.00/0.10
	*p*	1.0	1.0	0.54	1.0	1.0	1.0	0.54	0.28	1.0

*M*/*F* male/female, *R*/*P* regressive/progressive, *Seg*/*Nonseg* segmental/nonsegmental, *FH* family history, *FHAIDs* family history of auto-immune diseases, *p* value significant if **<** 0.05

Fischer Exact test: no significant associations were detected

**Table 6:** Relation of TLR4 genotypes versus demographic, clinical, and outcome data in vitiligo group 2 (above 18 years) patients

SNPs		*RS1927914*			*RS1927911*			*RS11536858*		
		*CC* *N*/*%*	*CT* *N*/*%*	*TT* *N*/*%*	*CC* *N*/*%*	*CT* *N*/*%*	*TT* *N*/*%*	*AA* *N*/*%*	*AG* *N*/*%*	*GG* *N*/*%*
Sex	M/F	0.12/0.13	0.21/0.17	0.67/0.71	0.27/0.22	0.58/0.60	0.15/0.18	0.24/0.26	0.60/0.59	0.17/0.14
	*P*	0.88	0.47	0.61	0.49	0.79	0.64	0.68	0.97	0.65
Course	Regressive/Progressive/stable	0.40/0.13/0.25	0.00/0.10/0.25	0.60/0.76/0.50	0.40/0.15/0.25	0.60/0.67/0.50	0.00/0.18/0.25	0.20/0.25/0.25	0.60/0.59/0.50	0.20/0.15/0.25
	*p*	0.25	0.48	0.39	0.33	0.75	0.54	0.97	0.93	0.84
Psychiatric trauma	+/−	0.09/0.30	0.08/0.11	0.61/0.79	0.09/0.21	0.74/0.62	0.17/0.17	0.17/0.28	0.70/0.55	0.13/0.17
	*p*	0.02	1.0	0.09	0.32	0.33	1.0	0.39	0.22	1.0
Alopecia	+/−	0.23/0.14	0.15/0.09	0.39/0.24	0.00/0.21	0.92/0.60	0.08/0.19	0.31/0.24	0.46/0.62	3/23.1
	*p*	0.42	0.62	0.28	0.11	0.03	0.45	0.73	0.29	0.42
FH of vitiligo	+/−	0.00/0.17	0.00/0.11	1.00/0.72	0.00/0.18	0.50/0.67	0.50/0.15	0.25/0.25	0.25/0.61	0.50/0.14
	*p*	1.0	1.0	0.57	1.0	0.60	0.13	1.0	0.29	0.12
FHAIDs	+/−	0.14/0.16	0.14/0.10	0.71/0.74	0.14/0.17	0.71/0.65	0.14/0.17	0.29/0.25	0.71/0.58	0.00/0.17
	*p*	1.0	0.56	0.89	1.0	0.74	1.0	1.0	0.49	0.59
Remission	+/−	0.18/0.10	0.09/0.15	0.73/0.75	0.18/0.15	0.63/0.75	0.20/0.10	0.23/0.30	0.64/0.45	0.13/0.25
	*p*	0.50	0.43	0.88	1.0	0.31	0.49	0.55	0.13	0.19
Type	Segmental/Nonsegmental	0.00/0.19	0.23/0.08	0.78/0.73	0.00/0.21	0.77/0.64	0.23/0.16	0.31/0.24	0.54/0.60	0.15/0.16
	*p*	0.11	0.13	0.77	0.11	0.35	0.69	0.73	0.67	1.0

*M*/*F* male/female, *R*/*P* regressive/progressive, *Seg*/*Nonseg* segmental/nonsegmental, *FH* family history, *FHAIDs* family history of auto-immune diseases, *p* value significant if **<** 0.05

Fischer exact test

Significant p value for the association between the CC genotype of rs1927914 and psychiatric trauma and between the CT genotype of rs1927911 and alopecia

Table 7 shows the combined genotypes of the studied 3 SNPs. Some of the combined genotypes have higher frequency in patients (*CCCTAA*, *CCCTAG*, *TTCTAA*, *TTCTTG*, *TTTTAA*, *TTCTAG*, *TTTTAG)*, while others have higher frequency in controls (*CTCCAA*, *CTCCAG*, *CTCTAA*, *CTCTAG*, *CTCTGG*, *TTCCAA*, *TTCCAG*, *TTCCGG*) and few have equal frequency in both patients and controls (*CCCCAG*, *CCCTGG*, *CTTTAA*, *CTTTAG*, and *TTTTGG*). No statistically significant relation has been found when patients and controls were compared against each other (*p* > 0.05).

**Table 7:** Distribution of TLR4 combined genotypes in vitiligo patients and healthy controls

TLR4 polymorphisms		Case (*N* = 100) *N* (%)	Control (*N* = 100) *N* (%)	OR (95% CI)	p/pc
COMBINED	*CCCCAG*	1	1	1.00 (0.16–16.21)	1.00/NS
	*CCCTAA*	2	1	2.02 (0.18–22.04)	0.57/NS
	*CCCTAG*	6	2	3.13 (0.62–15.89)	0.17/NS
	*CCCTGG*	1	1	1.00 (0.16–16.21)	1.00/NS
	*CTCCAA*	1	2	0.49 (0.04–5.55)	0.57/NS
	*CTCCAG*	2	4	0.49 (0.09–2.74)	0.42/NS
	*CTCTAA*	2	3	0.66 (0.11–4.03)	0.65/NS
	*CTCTAG*	6	9	0.65 (0.22–1.89)	0.42/NS
	*CTCTGG*	1	3	0.33 (0.03–3.19)	0.34/NS
	*CTTTAA*	1	1	1.00 (0.16–16.21)	1.00/NS
	*CTTTAG*	2	2	1.00 (0.14 –7.24)	1.00/NS
	*TTCCAA*	3	5	0.59 (0.14–2.53)	0.48/NS
	*TTCCAG*	8	11	0.70 (0.27–1.83)	0.74/NS
	*TTCCGG*	2	3	0.66 (0.11–4.03)	0.65/NS
	*TTCTAA*	11	10	1.11 (0.45–2.75)	0.82/NS
	*TTCTAG*	29	23	1.34 (0.72–2.85)	0.33/NS
	*TTCTGG*	8	7	1.16 (0.40–3.32)	0.79/NS
	*TTTTAA*	3	2	1.52 (0.25–9.27)	0.65/NS
	*TTTTAG*	8	5	1.65 (0.52–5.24)	0.39/NS
	*TTTTGG*	2	2	1.00 (0.14–7.24)	1.00/NS

*OR* odds ratio, *95*%*CI* 95% confidence interval. Significant *p*/pc values if **<** 0.05, *pc* Bonferroni corrected *p* value (number of comparison × *p* value). *NS* non-significant

Fisher’s exact: variability in combined genotype with no statistically significant relation

**Fig. 2:**
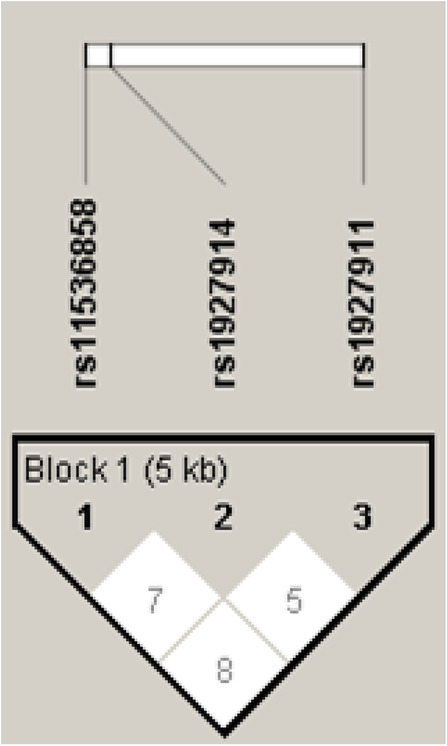
Linkage disequilibrium (LD) for the selected SNPs in cases and controls (using Haploview software analysis) [10]. The colored squares represent the numerical estimation of *D*′ value. The *D*′ value for LD between *rs1536858* and *rs1927914* was 0.071, between *rs1536858* and *rs1927911* was 0.089, and between *rs1927914* and *rs1927911* was 0.051 respectively

The VIDA score was done for vitiligo patients. Table 8 shows the relation between studied the selected SNPs and VIDA score. No statistically significant relations were observed (*p* > 0.05) when each SNP was compared against the scale of the VIDA score. Table 9 shows the frequency of haplotypes for TLR4 SNPs in both patients and controls. The *ATT* haplotype shows the highest frequency in patients while the *ATC* shows the least frequency in the same group. In control group, the *GCT* haplotype shows the highest frequency while the *GTC* and *GCC* shows the least frequency. On the other hand, no statistically significant relation has been detected when haplotypes frequencies were compared in both patients and controls (*p* > 0.05). The linkage disequilibrium (LD) of TLR4 *rs1927911*, *rs1927914*, and *rs11536858* polymorphisms was investigated. It was weak between different studied SNPs. The LD between *rs1536858* and *rs1927911* was D 0.071, between *rs1536858* and *rs1927911* was 0.089, and between *rs1927914* and *rs1927911* was D 0.051 (Fig. 2).

**Table 8:** Association of the TLR4 SNPs with the VIDA score of vitiligo

		*N*	Mean	Std. Deviation	Std. Error	95% Confidence interval for mean		*p* value*
						Lower bound	Upper bound	
*rs11536858*	0	22	2.0000	.43644	.09305	1.8065	2.1935	0.333
	1	13	1.8462	.55470	.15385	1.5110	2.1814	
	2	14	1.8571	.66299	.17719	1.4743	2.2399	
	3	15	2.0000	.65465	.16903	1.6375	2.3625	
	4	36	1.8333	.69693	.11616	1.5975	2.0691	
	Total	100	1.9000	.61134	.06113	1.7787	2.0213	
*rs1927914*	0	22	2.3636	.84771	.18073	1.9878	2.7395	0.237
	1	13	2.5385	.87706	.24325	2.0085	3.0685	
	2	14	2.7143	.61125	.16336	2.3614	3.0672	
	3	15	2.4667	.83381	.21529	2.0049	2.9284	
	4	36	2.7500	.60356	.10059	2.5458	2.9542	
	Total	100	2.5900	.73985	.07398	2.4432	2.7368	
*rs1927911*	0	22	1.9091	.52636	.11222	1.6757	2.1425	0.817
	1	13	2.0769	.64051	.17765	1.6899	2.4640	
	2	14	1.7857	.69929	.18689	1.3820	2.1895	
	3	15	1.9333	.59362	.15327	1.6046	2.2621	
	4	36	2.1667	.56061	.09344	1.9770	2.3564	
	Total	100	2.0100	.59450	.05945	1.8920	2.1280	

*One-way ANOVA: No statistically significant relations were observed (*p* > 0.05) when each SNP was compared against the scale of the VIDA score

**Table 9:** The distribution of TLR4 haplotypes in patients and controls

Haplotype	Frequency	Case, Control Ratio counts	Case-control frequencies	Chi-square	*p* value
*ATT*	0.229	51.0 : 149.0, 40.7 : 159.3	0.255, 0.203	1.507	0.2197
*ACT*	0.213	40.6 : 159.4, 44.7 : 155.3	0.203, 0.224	0.256	0.6126
*GCT*	0.208	36.0 : 164.0, 47.1 : 152.9	0.180, 0.236	1.874	0.171
*GTT*	0.147	31.4 : 168.6, 27.5 : 172.5	0.157, 0.137	0.309	0.5781
*ACC*	0.068	13.9 : 186.1, 13.4 : 186.6	0.069, 0.067	0.0060	0.9359
*GTC*	0.057	14.0 : 186.0, 8.7 : 191.3	0.070, 0.043	1.321	0.2504
*GCC*	0.043	8.6 : 191.4, 8.7 : 191.3	0.043, 0.043	0.0010	0.9712
*ATC*	0.034	4.6 : 195.4, 9.2 : 190.8	0.023, 0.046	1.573	0.2098

Variation in TLR4 SNPs haplotype frequencies in both patients and controls. No statistically significant difference of haplotypes frequencies between both groups was reported (*p* < 0.05)

Table 10 shows the relation between different haplotypes with clinical and demographic data of selected patients’ group. The only significant relations were found between *ACC* haplotype and family history of AIDs (*p* 0.02) and between *ATT* haplotype and remission (*p* 0.02). No other significant relations were detected between the other haplotypes and different data (*p* > 0.05).

**Table 10:** Relation of TLR4 haplotypes versus demographic, clinical, and outcome data in vitiligo patients

		Haplotype							
		ACC	ACT	ATC	ATT	GCC	GCT	GTC	GTT
Sex	M/F	0.04/0.09	0.26/0.14	0.04/0.00	0.26/0.25	0.00/0.07	0.17/0.18	0.06/0.07	0.13/0.18
	*P*	0.45	0.14	0.20	0.89	0.12	0.96	1.00	0.96
Course	R/P	0.00/0.07	0.50/0.14	0.00/0.02	0.50/0.25	0.00/0.04	0.00/0.20	0.00/0.07	0.00/0.17
	*P*	1.00	0.05	1.00	0.34	1.00	0.59	1.00	0.58
Psychic trauma	NO/Yes	0.06/0.07	0.20/0.19	0.01/0.03	0.23/0.34	0.04/0.03	0.21/0.07	0.04/0.15	0.18/0.07
	*p*	1.00	0.91	0.45	0.25	1.00	0.14	0.07	0.23
Alopecia	NO/Yes	0.05/0.14	0.19/0.21	0.02/0.00	0.26/0.21	0.04/0.00	0.18/0.14	0.04/0.21	0.17/0.07
	*p*	0.25	1.00	1.00	1.00	1.00	1.00	0.06	0.46
FH vitiligo	NO/Yes	0.05/0.25	0.19/0.25	0.02/0.00	0.26/0.25	0.04/0.00	0.18/0.12	0.06/0.12	0.17/0.00
	*p*	0.09	0.65	1.00	1.00	1.00	1.00	0.45	0.35
FHAIDs	NO/Yes	0.04/0.33	0.19/0.22	0.02/0.00	0.28/0.00	0.04/0.00	0.19/0.00	0.06/0.11	0.14/0.33
	*p*	0.02	1.00	1.00	0.11	1.00	0.35	0.49	0.15
Remission	NO/Yes	0.08/0.06	0.18/0.21	0.00/0.03	0.40/0.17	0.02/0.04	0.16/0.19	0.02/0.09	0.11/0.19
	*p*	0.71	1.00	0.53	0.02	1.00	0.79	0.25	0.39
Type	Seg/Non-Seg	0.05/0.07	0.17/0.20	0.00/0.02	0.41/0.22	0.05/0.03	0.11/0.19	0.05/0.07	0.11/0.16
	*p*	1.00	1.00	1.00	0.12	0.53	0.73	1.00	0.73

*M*/*F* male/female, *R*/*P* regressive/progressive, *Seg*/*Nonseg* segmental/nonsegmental, *FH* family history, *FHAIDs* family history of auto-immune diseases, *p* value significant if < 0.05

Fischer exact test

Significant *p* value between ACC haplotype and family history of AID and between ATT haplotype and remission

## Discussion

To date, vitiligo is still recognized as a puzzling skin disease that involves different key players leading finally to melanocyte destruction [12]. Based on a systematic meta-analysis, vitiligo was reported as a highly prevalent disease in Africa [13]. Considering that Egypt is one of the largest countries in Africa, in terms of population density, we tried to shed light on genetic polymorphisms in such multifactorial common disease in our population rich locality, aiming to clarify the pathophysiology of vitiligo and hence, increase the possibility to improve the therapeutic modalities.

In vitiligo, the innate immunity runs a two-signal pathways mediated by two main groups of receptors: toll-like receptors (TLRs) and nod-like receptors (NLRs), that identify PAMPs expressed on different microbial surfaces [14]. Polymorphisms in NLRs have been described in patients with non-segmental vitiligo [15]. Different TLRs polymorphisms have been studied in vitiligo (discussed later) [16, 17].

TLRs recognize endogenous molecules, referred to as danger-associated molecular patterns (DAMPs) [18]. Among these DAMPs is the heat shock protein 70 (HSP70i) [19]. TLR4 is expressed on melanocytes and believed to react to endogenous heat-shock proteins and initiate autoimmunity [20] by stimulating dendritic cells (DCs) to present melanocyte-specific antigens to T cells in lymphoid tissues [21]. Activated DCs release a cascade of cytokines that stimulate keratinocytes and activated NK cells together with chemo-attracted melanocyte-specific CD8^+^ T cells leading to the T cell-mediated autoimmune destruction of melanocytes [22].

The TLR4 gene (*gene ID 7099*) is located at chromosome *9q33.1*, and its SNP *rs1927914* is located in the 5′ flanking region of the TLR4 gene [23], while *rs11536858* is located in the promotor region. Both may function either by affecting the binding affinity of transcription factors to the regulatory TLR region or by targeting the extracellular domain of TLR4 receptor which controls the receptor binding affinity to its ligands [24]. On the other side, *rs1927911* is located in intron 1 of TLR4 gene and it is not known either is located in a functional site or not. The variations in intron may affect the proper mechanism of mRNA transcription or splicing [25].

In literature, most of studied TLR4 SNPs in Egyptian population were *rs4986790* and *rs4986791*. Furthermore, they were studied in different disorders like pulmonary tuberculosis [26], colorectal cancer [27], familial Mediterranean fever [28], rheumatoid arthritis, and systemic lupus erythematosus [29]. In this research, we decided to test different TLR4 SNPs. The TLR4 SNPs *rs11536858*, *rs1927914*, and *rs1927911* were selected, for the reason that they have not been studied in Egyptians before, to our knowledge, despite being studied in other disorders and nationalities [5, 9].

In our study, TLR4 *rs11536858*, *rs1927914*, and *rs1927911* gene polymorphisms were examined in 100 Egyptian patients diagnosed with vitiligo versus 100 controls. Vitiligo cases and control groups were stratified according to age into 2 groups (group 1 below 18 years versus group 2 above 18 years), and according to gender. Correlating the genotypes of the selected TLR4 SNPs and the demographic and clinical data of the vitiligo patients revealed no significant associations in group 1 (below 18 years). On the other hand, the older age group (above 18 years) showed a significant association between the *CC* genotype of *rs1927914* and psychiatric trauma (*p* 0.02) and between the *CT* genotype of *rs1927911* and alopecia (*p* 0.03). Traks et al. 2015 studied the association of 30 SNPs from different TLRs in Estonian case-control samples (139 vs. 307 respectively) and showed significant associations between some TLR gene SNPs and susceptibility to vitiligo; the results were significant in TLR4 SNP *rs10759932* TLR4 which showed significant association with familial cases but not with sporadic cases [17]. In our study, we have not found such significant association in familial vitiligo cases. In our opinion, the disagreement could be attributed to the difference in number of studied cases, in addition to the different ethnic backgrounds of the studied groups.

Concerning the distribution of the genotypes and alleles of the selected SNPs in both patients and controls, the only significant relation (even after application of Bonferroni correction) was the *CT* genotype of *rs1927914* being higher in control group (OR = 0.35, CI = 0.16–0.76, *p* = 0.01 and pc = 0.03). No other significant relations have been found for other alleles and genotypes. A study, in 2013, that included similar groups to ours in terms of numbers (100 vitiligo patients versus 100 controls) on TLR2 gene *Arg753Gln*, TLR4 gene *Asp299Gly*, and *Thr399Ile* polymorphisms, showed that the distribution of TLR4 *Asp299Gly* genotype was significantly higher in the patient group (10%) than in the control group (%2) (*p* < 0.05). The TLR4 *Thr399Ile* distribution did not show any difference in both vitiligo and healthy groups [16]. Again, the difference here could be attributed to the different ethnic backgrounds as it was conducted on Turkish population.

The previous two studies correlated polymorphisms with vitiligo activity, disease onset, and age of appearance, but did not correlate them with other risk factors or treatment protocols. In our study, TLR4 gene genotypes showed no significant differences regarding gender, segmental and non-segmental varieties of vitiligo, achieved remission after phototherapy. The statistically significant association with positive history of psychiatric trauma (in the older age group) supports the hypothesis of the association between stress and autoimmune diseases [30]. The other statistically significant association with alopecia supports the evidence of association of alopecia with vitiligo as one of the autoimmune diseases of the skin that share common pathways of pathogenesis [31].

Polymorphisms of TLR4 have been associated with autoimmune diseases in the skin or elsewhere. A study by Garcia-Rodriguez et al. 2013 showed a clear increase in TLR4 gene expression in patients with psoriasis [32]. A meta-analysis for the association of TLR4 polymorphism with the susceptibility to develop systemic lupus erythematosus was done in 2016 [33]. Another study by Alzolibani and colleagues, in 2016, alopecia areata patients expressed several TLRs (namely TLRs 3, 7, 8, and 9), though TLR4 expression was not significantly different from that of the control group [34]; this study paved the way for further investigation about the role of TLR4 in autoimmune diseases of the skin.

Other systemic diseases like Crohn’s disease and ulcerative colitis showed increased TLR4 expression [35]. Same results were applied to chorionic plate inflammation [36]. In asthma, genetic analysis of TLR4 *rs1927914* was found to be linked to asthma severity [23]. TLR4 gene polymorphisms were also found to be associated with Behcet’s disease [37].

In our study, we did the VIDA score for all vitiligo cases. Active vitiligo involves either expansion of existing lesions or appearance of new lesions. Grading is from 1 to 4 based on stability of vitiligo lesions in relation to duration. A low VIDA score indicates less activity [38]. Results showed no statistically significant relations were observed (*p* > 0.05) when each SNP was compared against the scale of the VIDA score. This comes in agreement with Karaca et al. (2013) who reported no significant association of TLR4 expression with vitiligo surface area [16].

Other interesting findings in our study are that some haplotypes appeared to be risky (namely the *ATT* haplotype) as it showed the highest frequency in patients, while the *ATC* shows the least frequency in the same group. In control group, the *GCT* haplotype shows the highest frequency (it seems to be protective haplotype) while the *GTC* and *GCC* shows the least frequency. Significant relations were found between *ACC* haplotype and family history of AIDs and between *ATT* haplotype and remission after phototherapy. The association with family history of AID is supported by the finding of several loci in vitiligo that are shared with other autoimmune disorders, such as thyroid disease, type 1 diabetes, and rheumatoid arthritis [39]. In concordance with the second finding, a theory that the Lack of TLR4 activity is associated with resistance to ultraviolet B (UVB)-induced immunosuppression was suggested by some authors [40], and explained by the fact that TLR4 is required for the generation of IL-10-secreting T regulatory cells involved in ultraviolet-induced immunosuppression [41]. A recent study proved that TLR4-mediated inflammation may potentiate the effects of UVB and even increase the incidence of UVB-induced skin cancers. Accordingly, inhibition of TLR4-mediated immune reactions could improve the therapeutic effects of UVB [42].

## Conclusions

Our findings support that innate immunity has significant role in pathogenesis of vitiligo. Targeting the innate immune receptors by immunotherapy may serve as novel therapeutic strategy for vitiligo. The significant relations between *ACC* haplotype and family history of AIDs emphasize the importance of the recommended protective healthy lifestyle for individuals with family history of autoimmune disease. Since the genetic etiological causes cannot be modified, a modification in environmental factors like (weight, stress, diet, and home environment) can be encouraged in order to protect from the autoimmune diseases. Our study is limited by the small population size and methodological limitations of PCR-RFLP, so more researches are recommended to confirm the present findings in Egyptian population and to clarify the mechanism of modulation of the function of TLR4 gene by various SNPs.

## Data Availability

The datasets used and/or analyzed during the current study are available from the corresponding author on reasonable request.

## Abbreviations

AIDs: Autoimmune diseases
APCs: Antigen-presenting cells
DAMPs: Danger-associated molecular patterns
FH: Family history
HSP70i: Inducible heat shock protein 70
LD: Linkage disequilibrium
NK: Natural killer
NLRs: Nod-like receptors
PAMPs: Pathogen-associated molecular patterns
PCR: Polymerase chain reaction
RFLP: Restriction fragment length polymorphism
SD: Standard deviation
SNP: Single-nucleotide polymorphism
TLR: Toll-like receptors
UVB: Ultraviolet B
VIDA: Vitiligo disease activity
